# Supplementary material for: Monitoring Functional Posttranslational Modifications Using a Data‐Driven Proteome Informatic Pipeline
Source: Proteomics. 2025 Mar 18;25(8):e202400238. doi: 10.1002/pmic.202400238 (PMC12019915; doi:10.1002/pmic.202400238)
Supplement: Supplementary file 1 — Supporting Information [file PMIC-25-e202400238-s004.docx]

## Supplementary data 5

**Monitoring Functional Post-Translational Modifications Using a Data-Driven Proteome Informatic Pipeline Based on PEIMAN2**

Payman Nickchi^1^, Uladzislau Vadadokhau^2^, Mehdi Mirzaie^3^, Marc Baumann^2^, Amir Ata Saei*^4^, Mohieddin Jafari*^2^

^1^Department of Statistics, University of British Columbia, Vancouver, Biritish Columbia, Canada

^2^Medicum, Department of Biochemistry and Developmental Biology, Meilahti Clinical Proteomics Core Facility, University of Helsinki, Helsinki, Finland

^3^Department of Pharmacology, Faculty of Medicine & Helsinki Institute of Life Science, University of Helsinki, Helsinki, Finland

^4^Department of Microbiology, Tumor and Cell Biology, Karolinska Institutet, Stockholm, Sweden

### Supplementary Figures

***Supplementary Figure 1:*** *PTM frequency treemaps for eight popular organisms from diverse taxonomic branches of life.*


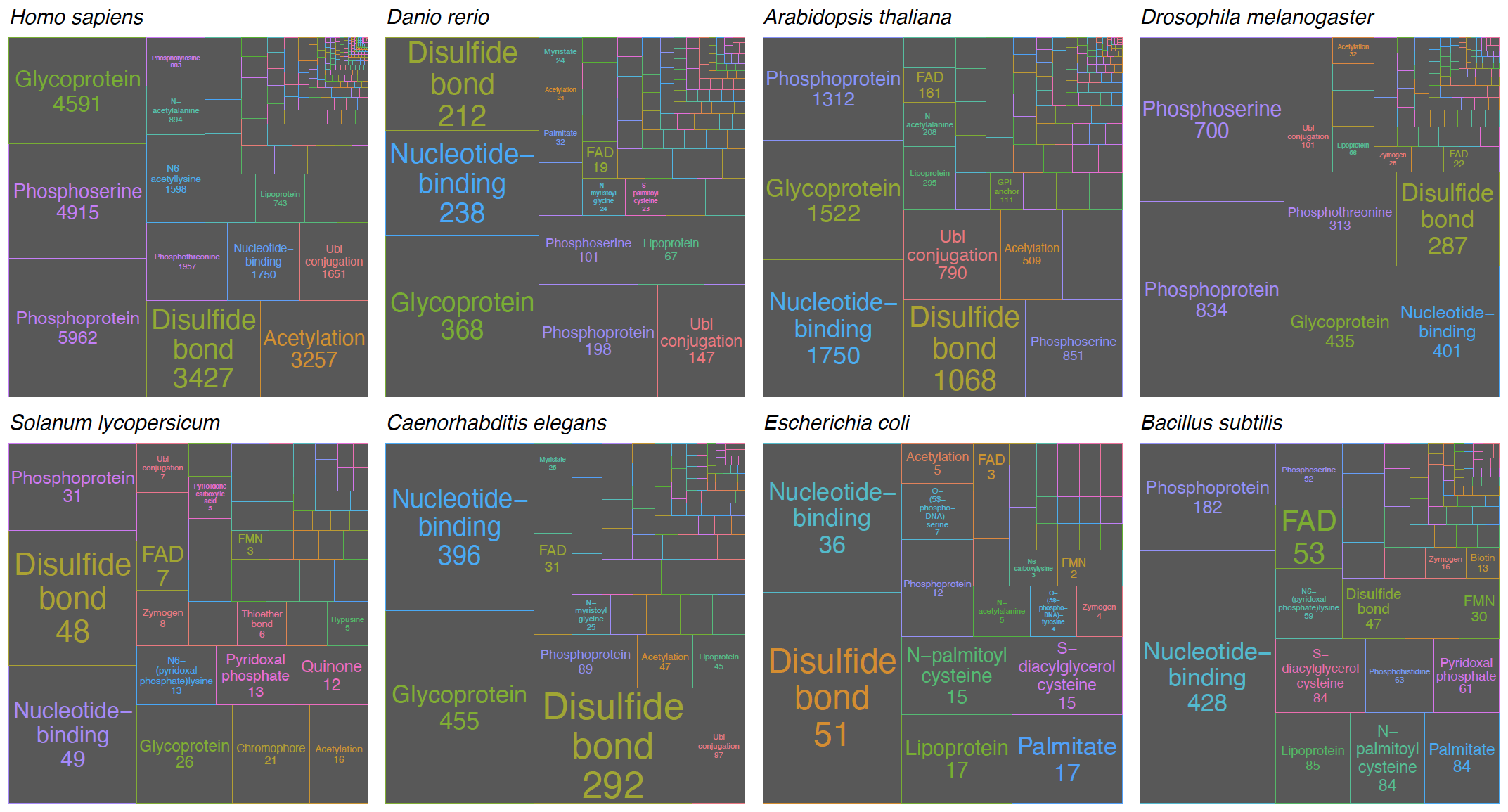


***Supplementary Figure 2:*** *The t-SNE plots based on PTM profiles in four super kingdoms of life, i.e., Archaea, Bacteria, Eukaryota and Viruses (panels A-D). Each dot in the plots presents one organism. The red and grey colors indicates if the point (organism) belongs to corresponding super kingdom of life or not. Note that we anticipate a more uniform distribution of viruses across all three of the other phyla of life (Panel D).*


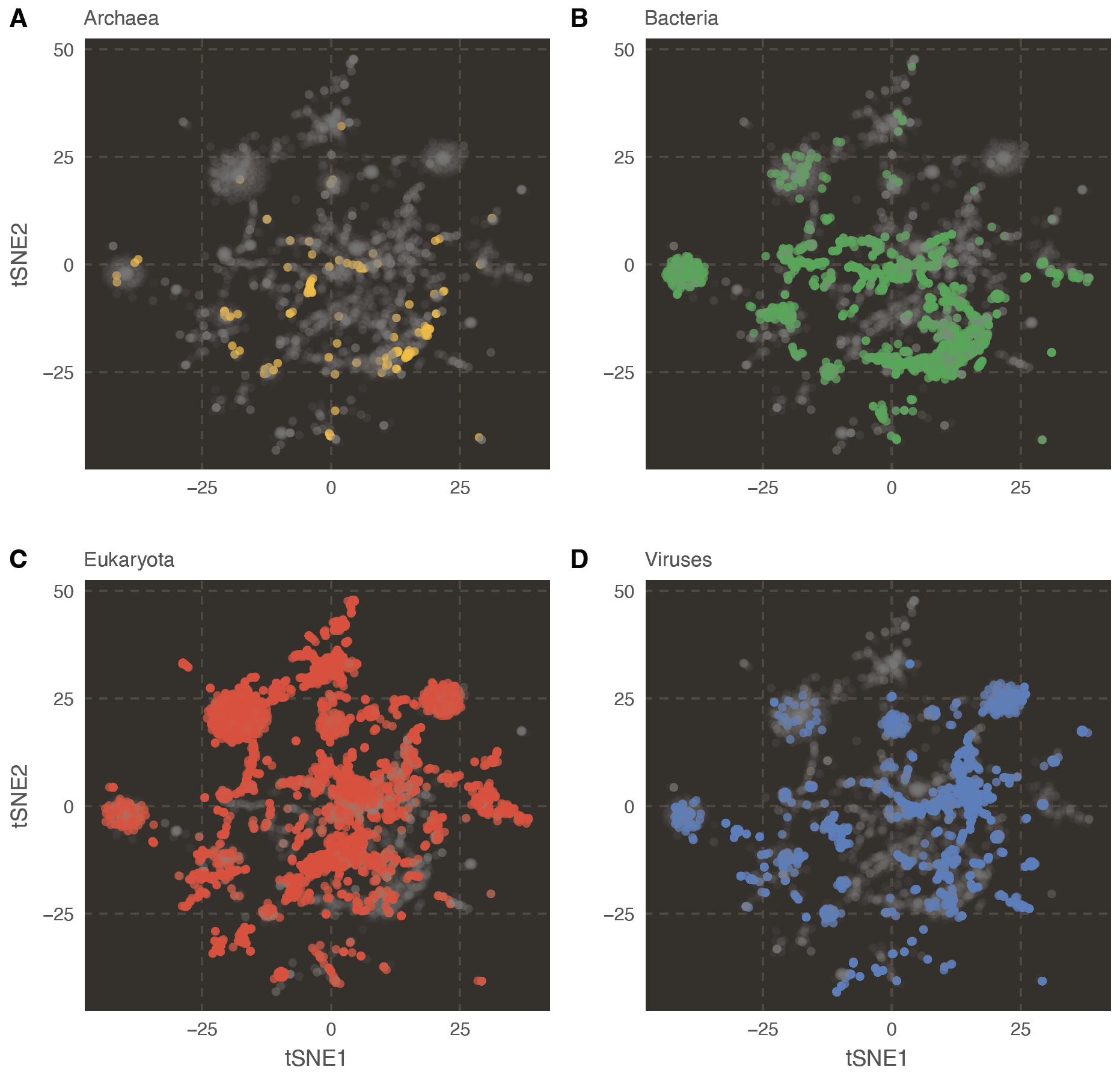


*
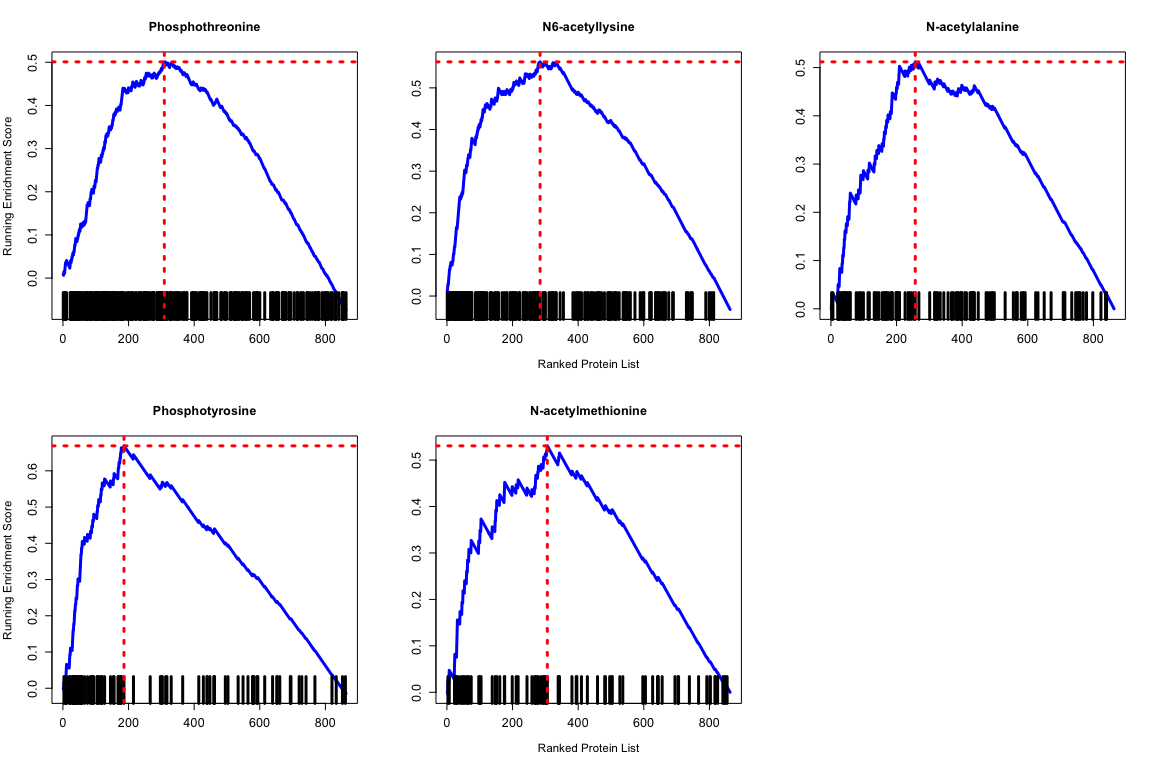
****Supplementary Figure 3:*** *The running score plot of the first top five PTMs identified on differentially expressed proteins upon dasatinib treatment. The x-axis is the ranked protein based on their score and the y-axis is their enrichment score. The rug in the x-axis indicates the proteins with the corresponding PTM. The position of maximum running enrichment score is denoted by a red dashed line.*


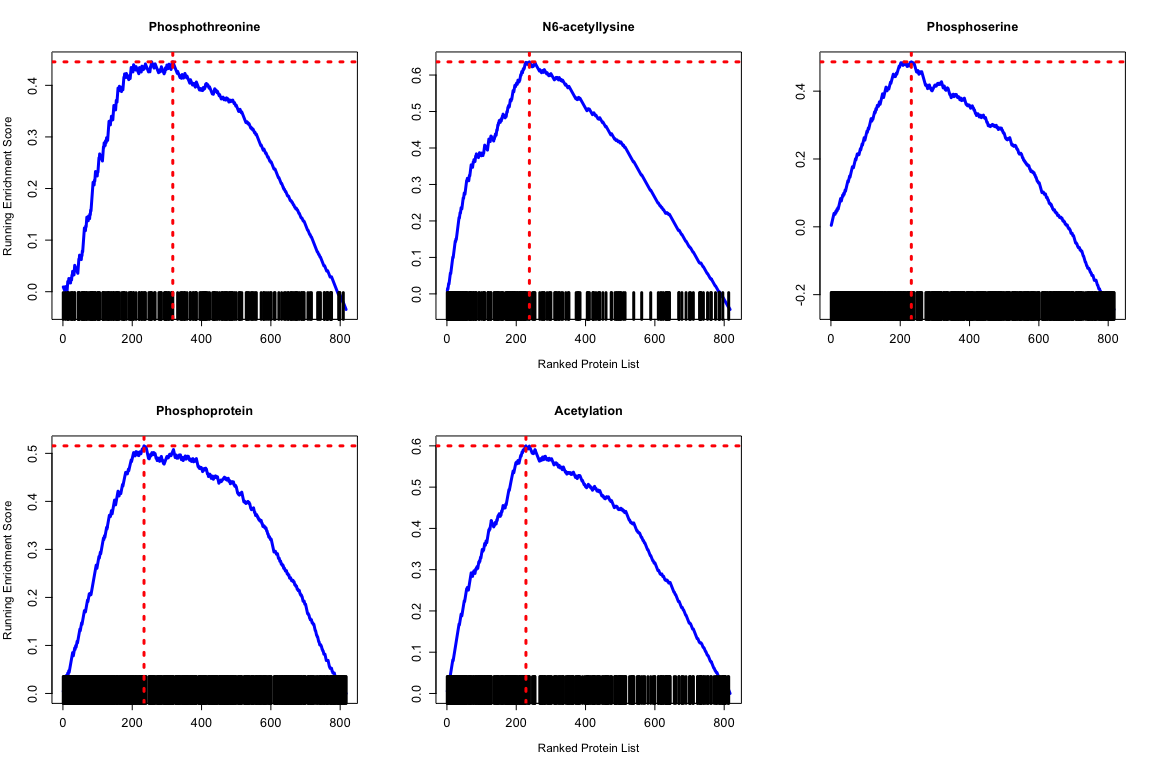
***Supplementary Figure 4:*** *The running score plot of the first top five PTMS identified on differentially expressed proteins upon staurosporine treatment. The x-axis is the ranked protein based on their score and the y-axis is their enrichment score. The rug in the x-axis indicates the proteins with the corresponding PTM. The position of maximum running enrichment score is denoted by a red dashed line.*

***Supplementary Figure 5:*** *The modified peptides with probabilities. The modified peptides with all above-mentioned PTMs are listed separately upon dasatinib and staurosporine treatment. The x-axis is the four drug concentrations and the control, and the y-axis is the proportional abundance of the corresponding peptide compared to unmodified peptide (see Supplementary data 3 and 4 for more details).* *Note: Identical peptide sequences may appear with different phosphorylation site probabilities, representing varying confidence levels in site localization.*

***Supplementary data 1***. *A table of all identified proteins for dasatinib following PTM-centric proteome informatic pipeline. This file represents the raw output from MaxQuant search before undergoing any filtering for subsequent analysis.*

***Supplementary data 2.*** *A table of all identified proteins for staurosporine following PTM-centric proteome informatic pipeline. This file represents the raw output from MaxQuant search before undergoing any filtering for subsequent analysis.*

***Supplementary data 3.*** *A compiled table of all PTM-carrying peptides for dasatinib and calculated final fold changes that are plotted in Supp figure 5.*

***Supplementary data 4.*** *A compiled table of all PTM-carrying peptides for staurosporine and calculated final fold changes that are plotted in Supp figure 5.*

***Supplementary data 5:*** *This pdf file contains the Supplementary Figures 1-5.*
